# Response-Adapted Benefit of Postoperative Adjuvant Therapy Following Neoadjuvant Treatment in Resectable NSCLC: A Single-Center Retrospective Cohort Study           

**DOI:** 10.3390/cancers18060955

**Published:** 2026-03-15

**Authors:** Yanbo Wang, Weiran Zhang, Xin Wang, Han Zhang, Qiuqiao Mu, Jianyu Wang, Qingsheng Liu, Guotai Wang, Xin Li, Daqiang Sun

**Affiliations:** 1 Clinical School of Thoracic, Tianjin Medical University, Tianjin 300070, China; wangyb202402@163.com (Y.W.); mqqfighting@163.com (Q.M.); wangjianyu0536@163.com (J.W.); 16622082729@163.com (Q.L.); pymachinegun@163.com (G.W.); 2 Chest Hospital, Tianjin University, Tianjin 300222, China; wrzhang1986@126.com (W.Z.); pumpkin2018@tmu.edu.cn (H.Z.); 3 Department of Thoracic Surgery, Tianjin Chest Hospital, Tianjin 300222, China; pku_wangxin@163.com

**Keywords:** NSCLC, neoadjuvant chemoimmunotherapy, major pathological response, adjuvant therapy, event-free survival, response-adapted strategy

## Abstract

**Background**: Neoadjuvant immunochemotherapy improves pathological response in resectable non-small cell lung cancer (NSCLC), but the need and intensity of postoperative adjuvant therapy across different pathological response rate (PRR) strata remain uncertain. **Methods**: In this single-center retrospective cohort, 105 patients with resectable NSCLC received neoadjuvant platinum-based chemotherapy with or without PD-1/PD-L1 inhibitors followed by R0 resection. PRR was defined as 1—residual viable tumor fraction and categorized as 0–60%, 60–90%, and ≥90% (major pathological response, MPR). Postoperative strategies included no further therapy, chemotherapy alone, or immunotherapy ± chemotherapy. Event-free survival (EFS) was analyzed using Kaplan–Meier estimates, multivariable Cox models, and restricted cubic spline-based treatment × PRR interaction. **Results**: Deeper PRR was associated with lower ypT/ypN stage and improved EFS. In the PRR 0–60% subgroup, immunotherapy-containing adjuvant regimens were associated with better EFS, whereas chemotherapy alone did not outperform observation. In the PRR 60–90% and MPR strata, EFS curves for different postoperative strategies largely overlapped, and in MPR patients, hazard ratios were close to 1. Interaction modeling suggested that the absolute 3-year EFS benefit of immunochemotherapy peaked at intermediate PRR (≈60–80%) and diminished as PRR approached ≥90%. **Conclusions**: The robustness of these findings was further confirmed through a sensitivity analysis focusing on a homogeneous cohort of clinical stage II-III patients receiving adjuvant therapy. Among NSCLC patients treated with neoadjuvant systemic therapy, PRR is a strong prognostic marker and modulates the benefit of postoperative immunotherapy. These data support a response-adapted strategy, with adjuvant immunotherapy intensification in low-PRR patients and potential de-escalation or surveillance alone in MPR patients, warranting validation in prospective PRR-stratified trials.

## Simple Summary

Treatment before surgery is effective for resectable non-small cell lung cancer, but it remains uncertain whether all patients require further treatment after surgery. This study aimed to determine if the necessity of postoperative therapy depends on how well the tumor responded to the initial treatment. The authors found that patients achieving a major pathological response derived minimal benefit from additional immunotherapy, whereas those with a poor response achieved significantly better survival outcomes with adjuvant immunotherapy. These findings suggest that postoperative treatment should be tailored based on pathological response rather than using a uniform approach. This research supports a personalized strategy that could spare good responders from unnecessary side effects and financial costs while ensuring that high-risk patients receive the intensive therapy required to prevent disease recurrence.

## 1. Introduction

Neoadjuvant immunotherapy combined with chemotherapy has emerged as a new paradigm in the perioperative management of resectable non-small cell lung cancer (NSCLC). Several pivotal trials, including CheckMate-816 [1,2,3,4,5], NADIM II [6,7,8], and KEYNOTE-671 [9], have demonstrated the potential of neoadjuvant immunotherapy to increase pathological response (PR) rates and improve intermediate-term survival outcomes. However, there remains a lack of consistent evidence and clear recommendations regarding the necessity and optimal use of postoperative adjuvant therapy—particularly in the context of varying pathological response rates (PRRs). Most previous studies have focused primarily on preoperative efficacy endpoints such as major pathological response (MPR) and pathological complete response [10,11] (pCR), whereas postoperative adjuvant strategies are frequently influenced by treating physicians’ preferences, patient tolerance, postoperative risk profiles, and pathological characteristics. In real-world clinical practice, PRR has become one of the key determinants of adjuvant therapy decisions: patients achieving MPR or better are often regarded as a favorable-prognosis subgroup, and some of these individuals may forgo further systemic therapy; conversely, those with low PRR, who are at higher risk of relapse, are more likely to receive additional adjuvant treatment [12]. Accumulating evidence indicates that deeper pathological responses after neoadjuvant chemoimmunotherapy are strongly associated with improved event-free and overall survival, supporting the use of PRR/MPR as surrogate endpoints and potential anchors for tailoring perioperative treatment intensity.

In parallel, dedicated adjuvant trials have established postoperative systemic therapy as an effective strategy in selected patients, including adjuvant atezolizumab after chemotherapy (IMpower010) and adjuvant pembrolizumab (PEARLS/KEYNOTE-091), while adjuvant EGFR-TKI therapy has reshaped care for EGFR-mutant disease (CTONG1104) [13,14,15,16,17]. Recent systematic reviews and reconstructed or indirect comparisons further highlight ongoing uncertainty regarding the incremental value of routine adjuvant immunotherapy after neoadjuvant chemoimmunotherapy, underscoring the need for response-adapted perioperative strategies and real-world validation [18,19,20,21,22,23,24,25,26,27,28,29,30,31,32]. The rapid translation of immune checkpoint blockade from advanced-stage disease to the resectable setting has been driven by durable survival benefits observed in metastatic trials and the ensuing ‘chemoimmunotherapy revolution’ [28,33].

## 2. Methods

### 2.1. Study Design and Patient Population

This single-center retrospective cohort study included patients with resectable non-small cell lung cancer (NSCLC) who received neoadjuvant systemic therapy followed by curative-intent resection at Tianjin Chest Hospital between January 2019 and December 2024. Eligibility criteria were as follows: (1) histologically or cytologically confirmed NSCLC before surgery; (2) receipt of neoadjuvant platinum-based doublet chemotherapy with or without an immune checkpoint inhibitor; (3) completion of R0 resection; and (4) availability of complete records on neoadjuvant and postoperative adjuvant treatment, postoperative pathological assessment, and follow-up. These eligibility criteria were chosen to broadly reflect the patient populations enrolled in contemporary neoadjuvant and perioperative chemoimmunotherapy trials in resectable NSCLC [34,35].

**Figure 1:**
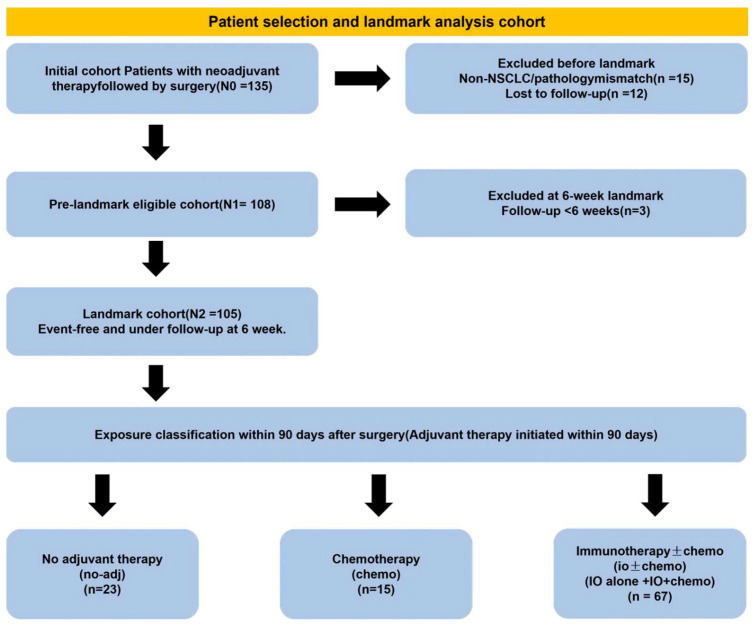
Patient selection and landmark analysis flow diagram. The diagram illustrates the patient inclusion process for the landmark analysis cohort and provides reasons for exclusion at each step (non-NSCLC/pathology mismatch, loss to follow-up, and follow-up <6 weeks at the 6-week landmark). Exposure was classified based on adjuvant therapy initiated within 90 days after surgery (no-adj, chemo, and IO ± Chemo). Abbreviations: NSCLC, non-small cell lung cancer; IO, immunotherapy.

Exclusion criteria included: (1) presence of distant metastasis (M1) before or after surgery; (2) intraoperative or postoperative determination of unresectability; (3) death or loss to follow-up within 6 weeks after surgery; and (4) substantially incomplete follow-up data. The overall design and data-selection strategy were consistent with prior real-world cohorts evaluating perioperative immunotherapy in NSCLC (Figure 1) [23,36].

Clinical data were independently extracted from the electronic medical record and imaging systems by two investigators, including demographic characteristics, tumor features, details of neoadjuvant and postoperative adjuvant therapy, pathological findings, and follow-up information. Discrepancies were resolved by a third investigator.

Because of the retrospective nature of this study, the requirement for written informed consent was waived. The study was approved by the institutional review board of Tianjin Chest Hospital and conducted in accordance with the principles of the Declaration of Helsinki.

### 2.2. Neoadjuvant Treatment and Pathological Assessment

Neoadjuvant regimens were determined by a multidisciplinary team (MDT) based on comprehensive clinical evaluation and mainly consisted of platinum-based doublet chemotherapy using third-generation cytotoxic agents, with or without a PD-1/PD-L1 inhibitor. Most patients received 2–4 cycles of neoadjuvant therapy, with minor adjustments in some cases according to clinical status, in line with contemporary neoadjuvant chemoimmunotherapy protocols in resectable NSCLC [1,18].

All patients underwent standard lobectomy [31,37,38] or pneumonectomy with systematic lymph node dissection 4–8 weeks after completion of neoadjuvant therapy, consistent with surgical practice in recent neoadjuvant immunotherapy studies. Postoperative pathological specimens were independently reviewed by two thoracic pathologists who were blinded to clinical information. The pathological response rate (PRR) was defined as the complement of the proportion of residual viable tumor cells within the tumor bed (PRR = 1—residual viable tumor fraction), according to quantitative immune-related pathologic response criteria [39] proposed for neoadjuvant immunotherapy. Based on published evidence supporting major pathological response (MPR, approximately ≥90% tumor regression) as a clinically meaningful surrogate endpoint [40], PRR was categorized a priori into three groups—0–60%, 60–90%, and ≥90% (MPR)—to characterize the depth of pathological response to neoadjuvant therapy [34,39].

### 2.3. Postoperative Adjuvant Treatment Strategies

Postoperative adjuvant treatment was determined by the MDT according to postoperative pathological results, patient performance status, and estimated risk of recurrence. According to the actual treatment delivered, postoperative adjuvant strategies were classified into three groups: (1) no further treatment (no-adj); (2) chemotherapy alone (chemo); and (3) immunotherapy with or without chemotherapy (IO ± Chemo). Patients receiving immune monotherapy were grouped into the “immunotherapy ± chemotherapy” category to maintain consistency and clinical interpretability. These categories mirror major patterns of postoperative systemic management in recent perioperative immunotherapy trials and real-world cohorts.

To minimize bias introduced by inter-patient variability in the timing of treatment initiation, a 90-day exposure window was defined. Patients who initiated and completed the planned adjuvant regimen within 90 days after surgery were assigned to the corresponding exposure group; those who did not start adjuvant therapy within this window were classified as having received no further treatment. To reduce immortal-time bias, a landmark analysis was performed at t = 6 weeks; patients who experienced an event or failed to achieve R0 resection before this landmark were excluded from the primary analysis. The choice of exposure window and landmark strategy was informed by methodological considerations and by the design of contemporary perioperative immunotherapy studies in early-stage NSCLC [41,42,43].

### 2.4. Endpoints and Follow-Up

The primary endpoint was event-free survival (EFS), defined as the interval from the date of curative-intent surgery to the occurrence of any of the following events: (1) locoregional recurrence or distant metastasis; (2) development of an unresectable second primary lung cancer; or (3) death from any cause. Patients without events were censored at the date of last follow-up. This definition is consistent with that adopted in recent perioperative immunotherapy trials in resectable NSCLC. Follow-up information was obtained from outpatient visit records, inpatient medical records, and telephone interviews. The follow-up cutoff date was 31 December 2024 [1,9,41,42,44].

All analyses were conducted on a complete-case basis. In these analyses, the control group was defined as patients who received either no postoperative adjuvant therapy or adjuvant chemotherapy alone. The extent of missingness for study variables was assessed, and the overall missing rate in this cohort was <5%; therefore, multiple imputation was not performed.

### 2.5. Statistical Analysis

All statistical analyses were performed using R software (version 4.5.1). Continuous variables are summarized as mean ± standard deviation or median (interquartile range), as appropriate, and compared between groups using the t-test or Mann–Whitney U test. Categorical variables are presented as counts and percentages and compared using the χ^2^ test or Fisher’s exact test.

For survival analyses, Kaplan–Meier methods were used to estimate EFS, and differences between groups were assessed using the log-rank test. Pre-specified subgroup analyses were conducted according to PRR categories (0–60%, 60–90%, and ≥90%), within which EFS was compared among the three postoperative strategies: no-adj, chemo, and IO ± Chemo.

To evaluate the independent associations between clinically relevant variables and EFS, multivariable Cox proportional hazards models were constructed, including covariates with clear clinical relevance and those commonly used in perioperative prognostic models for NSCLC. Results are reported as hazard ratios (HRs) with 95% confidence intervals (95% CIs). The proportional hazards assumption was assessed using Schoenfeld residuals.

PRR was further modeled as a continuous variable using restricted cubic splines (RCS), with the number of knots prespecified within 3–5 and placed at recommended empirical percentiles to balance flexibility and parsimony, and an interaction model was constructed to evaluate the effect modification between postoperative treatment (immunotherapy ± chemotherapy vs. control) and PRR [31]. This model was used to estimate 3-year EFS across the spectrum of PRR and to characterize the pattern of treatment benefit (immunotherapy ± chemotherapy vs. control) as a function of PRR. Likelihood ratio tests were used to compare models with and without the interaction term in order to assess the overall interaction effect.

Unless otherwise specified, a two-sided *p* value < 0.05 was considered statistically significant.

## 3. Results

### 3.1. Baseline and Pathological Characteristics

A total of 105 eligible patients with NSCLC underwent curative-intent resection following neoadjuvant systemic therapy. The median age was 66 years (IQR 60–71), and 81.0% were male. Squamous cell carcinoma was the predominant histology (73.3%). The majority of patients presented with clinically locally advanced disease, with 60.0% having stage III and 33.3% having stage II disease at baseline.

Neoadjuvant immunochemotherapy was administered to 86 patients (81.9%), while 19 (18.1%) received chemotherapy alone. Most patients (80.0%) completed 2–3 cycles of neoadjuvant therapy. Lobectomy was the standard surgical procedure (67.6%), and video-assisted thoracoscopic surgery (VATS) was the primary approach (77.1%). Postoperative pathology revealed ypT1 and ypT3 as the most frequent tumor categories (38.1% each), and 59.0% of patients achieved ypN0 status.

Table 1 Baseline clinicopathologic features of all enrolled patients are summarized according to pathologic response rate (PRR) after neoadjuvant therapy: low response (PRR 0–60%), intermediate response (PRR 60–90%), and major pathologic response (MPR; PRR ≥ 90%). Continuous variables are presented as median (interquartile range), and categorical variables as the number of patients (percentage). PRR, pathologic response rate; MPR, major pathologic response; IQR, interquartile range.

### 3.2. Association Between Pathological Response and Clinicopathologic Features

Patients were stratified into three groups based on pathological response rate (PRR): 0–60% (*n* = 55), 60–90% (*n* = 15), and MPR (≥90%, *n* = 35). Baseline tumor size and clinical stage were comparable across groups (*p* > 0.05). However, male sex was significantly more frequent in the MPR group (94.3%) compared with the PRR 0–60% (76.4%) and PRR 60–90% (66.7%) groups (*p* = 0.034).

Patients treated with neoadjuvant immunochemotherapy achieved deeper pathological responses compared with those receiving chemotherapy alone (*p* = 0.048). As expected, higher PRR was associated with significant pathological downstaging: the proportion of ypT1 disease increased from 30.9% in the PRR 0–60% group to 48.6% in the MPR group, and the rate of ypN0 status increased from 52.7% to 68.6%, respectively. Postoperative adjuvant strategies were distributed similarly across strata (*p* = 0.326), although the MPR group had a numerically higher proportion of patients receiving immunochemotherapy (74.3%).

Table 2 Neoadjuvant treatment characteristics and key perioperative pathological outcomes are summarized overall and across strata of pathologic response rate (PRR 0–60%, PRR 60–90%, and major pathologic response [MPR; PRR ≥ 90%]). Variables include neoadjuvant regimen and number of cycles, type of pulmonary resection and surgical approach, postoperative ypT, ypN and ypTNM stage, tumor and nodal downstaging, and postoperative adjuvant regimen. Data are presented as number of patients with corresponding percentages, and *p* values refer to comparisons across PRR strata. PRR, pathologic response rate; MPR, major pathologic response; yp, post-treatment pathologic.

### 3.3. PRR-Stratified Comparison of EFS (Kaplan–Meier Curves)

**Figure 2:**
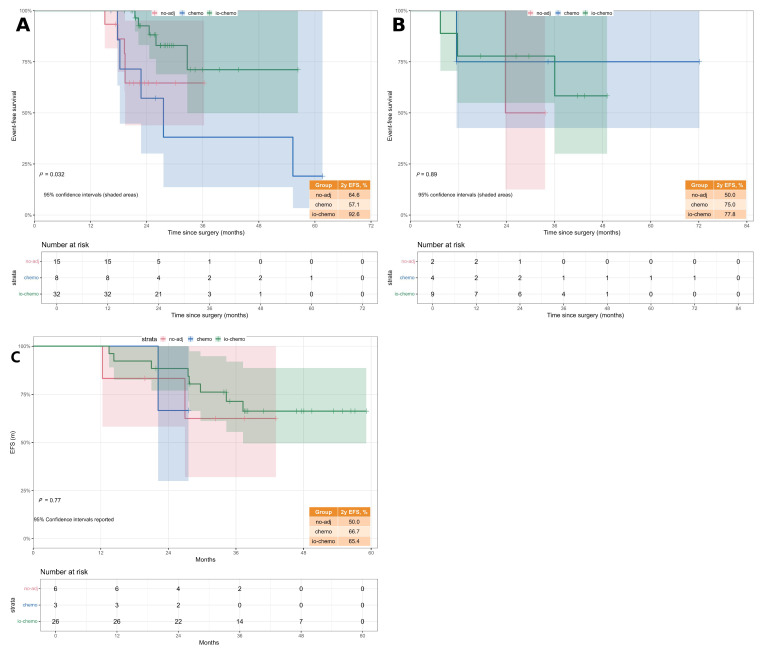
Event-free survival according to postoperative adjuvant strategy across pathologic response strata. (**A**) Kaplan–Meier curves of event-free survival (EFS) from the date of surgery in patients with a low pathologic response rate (PRR 0–60%), stratified by postoperative adjuvant regimen: no adjuvant therapy, chemotherapy alone, or immunochemotherapy (IO ± Chemo). (**B**) Corresponding EFS curves in patients with an intermediate pathologic response (PRR 60–90%). (**C**) EFS curves in patients achieving major pathologic response (MPR; PRR ≥ 90%). Shaded areas indicate 95% confidence intervals for each treatment group. Numbers at risk over time are displayed in the lower panels, and inset tables summarize the estimated 3-year EFS for each adjuvant strategy within each PRR stratum. EFS, event-free survival; IO ± Chemo, immunotherapy ± chemotherapy; no-adj, no adjuvant therapy.

Kaplan–Meier survival analysis revealed that the clinical efficacy of postoperative adjuvant therapy is intrinsically linked to the depth of pathological response, with the incremental benefit of treatment progressively diminishing as pathological regression improves. In the low-response stratum (PRR 0–60%; Figure 2A), a significant survival advantage was observed (*p* = 0.032), where adjuvant immunochemotherapy (io-chemo) provided robust protection, yielding a 2-year EFS rate of 92.6%, compared to 57.1% for chemotherapy and 64.6% for observation. However, this therapeutic superiority began to attenuate in the intermediate stratum (PRR 60–90%; Figure 2B); although active treatments maintained a numerical lead over observation (2-year EFS: 77.8% and 75.0% vs. 50.0%), the statistical difference between cohorts became non-significant (*p* = 0.89). This trend of convergence culminated in the major pathological response (MPR) subgroup (PRR 90–100%; Figure 2C), where survival curves for immunochemotherapy (65.4%) and chemotherapy (66.7%) essentially coalesced (*p* = 0.77). These findings suggest that while intensified adjuvant immunotherapy is critical for patients with poor pathological regression, its marginal utility is minimal once a deep pathological remission is achieved, potentially identifying a candidate population for treatment de-escalation [45,46].

### 3.4. Sensitivity Analysis of a Homogeneous Subgroup

To address potential selection bias, a supplementary analysis was performed on patients with clinical stage II or higher who received adjuvant therapy. The results remained consistent: patients with poor pathological response (ypIIA/IIIA) exhibited significantly higher recurrence risks, and intensified adjuvant IO + Chemo showed a clear trend toward superior EFS (*p* = 0.011, Supplementary Figures S1 and S2).

### 3.5. Multivariable Cox Regression in the Overall Cohort

A multivariable Cox proportional hazards model was conducted to identify independent predictors of event-free survival (EFS), adjusting for potential confounders including age, sex, ypTNM stage, histologic subtype, neoadjuvant regimen, and adjuvant treatment strategy.

**Figure 3:**
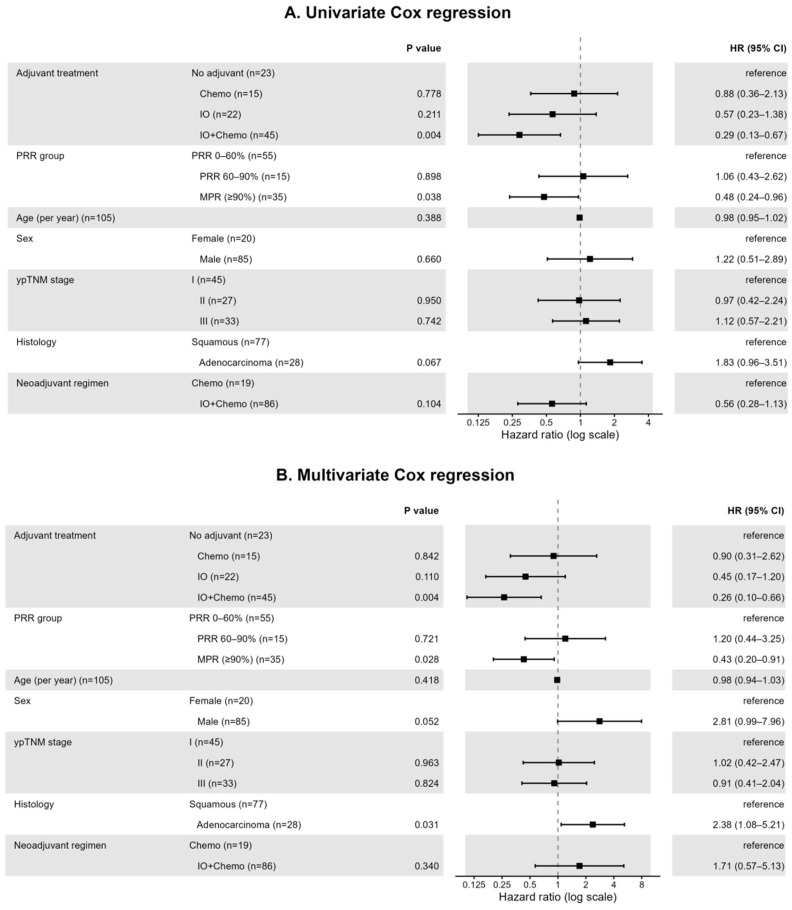
Forest plots of Cox proportional hazards regression analysis for Event-Free Survival (EFS). (**A**) Univariate Cox regression analysis. The forest plot displays unadjusted hazard ratios (HRs) for baseline characteristics and treatment factors. (**B**) Multivariate Cox regression analysis. The forest plot illustrates independent predictors of EFS after adjusting for age, sex, ypTNM stage, histologic subtype, neoadjuvant regimen, adjuvant treatment, and PRR group.

Pathological response rate (PRR) was confirmed as a significant independent prognostic factor. Patients achieving a Major Pathological Response (MPR, ≥90%) experienced a substantial survival benefit, with a 57% reduction in the risk of recurrence or death compared with the low-response reference group (PRR 0–60%) (HR 0.43; 95% CI 0.20–0.91; *p* = 0.028). This underscores the independent predictive value of deep pathological response for long-term outcomes (Figure 3).

Histologic subtype also emerged as an independent risk factor. Patients with adenocarcinoma exhibited a significantly worse prognosis compared with those with squamous cell carcinoma (HR 2.38; 95% CI 1.08–5.21; *p* = 0.031), suggesting a more aggressive clinical course in the adenocarcinoma subgroup.

Additionally, the analysis reaffirmed the robust protective effect of adjuvant immunotherapy combined with chemotherapy (IO ± Chemo), which was associated with a markedly lower risk of EFS events compared with no adjuvant treatment (HR 0.26; 95% CI 0.10–0.66; *p* = 0.004) [10,11,31].

### 3.6. Treatment × PRR Interaction Analysis and 3-Year EFS Prediction

**Figure 4:**
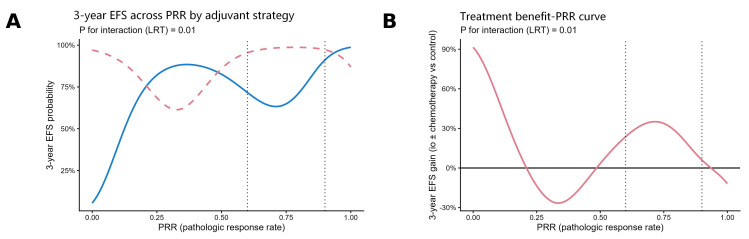
Association between pathologic response rate and 3-year event-free survival (EFS) and treatment benefit of postoperative immunochemotherapy. (**A**) Predicted 3-year EFS across the continuum of pathologic response rate (PRR) for patients receiving control therapy (solid blue line; no adjuvant therapy or chemotherapy alone) versus immunotherapy ± chemotherapy (dashed pink line), based on a weighted Cox model with restricted cubic splines. Vertical dotted lines indicate the prespecified PRR thresholds of 60% and 90%. (**B**) Corresponding treatment benefit–PRR curve showing the absolute 3-year EFS gain of immunotherapy ± chemotherapy over control (pink line). Values above 0% indicate a net benefit of immunotherapy-based adjuvant treatment, whereas values below 0% suggest harm or lack of benefit; vertical dotted lines again mark the 60% and 90% PRR cutoffs. PRR, pathologic response rate; EFS, event-free survival; io, immunotherapy.

To further characterize the relationship between response depth and adjuvant treatment efficacy, we performed an interaction analysis modeling PRR as a continuous variable using restricted cubic splines (RCSs). This model revealed a non-linear distribution of therapeutic benefit (Figure 4). The absolute 3-year EFS benefit of adjuvant immunotherapy (versus control) peaked in patients with intermediate pathological response (PRR ≈ 60–80%). Conversely, as PRR approached and exceeded 90% (MPR), the incremental survival benefit diminished progressively, with hazard ratios approaching 1.0 and confidence intervals crossing the null value [43,45]. This continuous modeling aligns with the categorical observations, confirming that the magnitude of adjuvant treatment benefit is dependent on the depth of prior pathological response [5,10,11].

## 4. Discussion

In this real-world cohort study, we challenged the conventional “one-size-fits-all” adjuvant treatment paradigm by delineating a distinct “gradient of therapeutic benefit,” demonstrating that the efficacy of postoperative immunotherapy is intrinsically linked to the depth of pathological response. First and foremost, our multivariable analysis established Pathological Response Rate (PRR)—specifically the achievement of a Major Pathological Response (MPR)—as a robust independent prognostic factor for event-free survival (HR 0.43; *p* = 0.028). This finding, which persists even after adjusting for adjuvant treatment intensity and other confounders, underscores that the depth of pathological regression serves as a surrogate for the tumor’s intrinsic biological sensitivity to immunotherapy and its metastatic potential, thereby validating PRR as a critical biomarker for risk stratification in the adjuvant setting.

Regarding the methodological concerns of treatment heterogeneity, it is worth noting that our supplementary sensitivity analysis—restricted to patients with clinical stage II-III who received postoperative therapy—yielded results highly congruent with our primary findings. This reinforces the notion that ypTNM stage [47,48], reflecting the residual tumor burden after neoadjuvant treatment, is a critical determinant for tailoring adjuvant strategies. Specifically, for those who fail to achieve deep pathological remission (Non-MPR or higher yp stage), the transition to intensified adjuvant regimens such as IO + Chemo may be necessary to overcome their inherently higher risk of recurrence.

Building upon this prognostic distinction, our results may support a risk-adapted stratified treatment strategy that moves beyond generic guidelines. For patients achieving MPR (PRR ≥ 90%), we observed a potential “ceiling effect” where survival curves for adjuvant chemotherapy, immunochemotherapy, and observation largely overlapped [49,50]. This implies that once the primary tumor and micrometastases are effectively eradicated by neoadjuvant therapy, the incremental survival gain from further postoperative intensification could be marginal. Consequently, for fit patients who have achieved deep pathological remission, treatment de-escalation—such as considering the omission of adjuvant immunotherapy or adopting a rigorous “watch-and-wait” approach—could be discussed as a potentially rational option to spare patients from unnecessary immune-related toxicities and financial burden without necessarily compromising long-term oncologic outcomes. Conversely, the therapeutic imperative differs sharply for patients with incomplete responses. In the low-response subgroup (PRR 0–60%), chemotherapy alone failed to arrest early disease recurrence, yielding outcomes numerically inferior even to observation, likely reflecting the selection of chemo-resistant clones. However, the addition of adjuvant immunotherapy significantly restored survival benefits (2-year EFS 92.6%) [35]. Thus, for these high-risk “non-responders,” maintaining or escalating adjuvant immunotherapy remains essential to control residual disease, marking a clear divergence from the de-escalation strategy proposed for the MPR population.

It is crucial, however, to interpret these findings within the context of specific limitations, particularly regarding sample size and selection bias. A seemingly paradoxical finding in our Kaplan–Meier analysis was the lower numerical survival rate in the untreated MPR subgroup. Rather than indicating a failure of the observation strategy itself, this is likely attributable to ‘negative selection bias’ inherent to real-world retrospective cohorts. To address this, our supplementary sensitivity analysis restricted to a homogeneous cohort (cStage II-III receiving adjuvant therapy) confirmed that the predictive value of ypTNM and PRR remains robust, and the survival benefit of intensified adjuvant IO + Chemo (*p* = 0.011) persists even after correcting for these confounders. Patients achieving MPR who opted out of adjuvant therapy (*n* = 6) often did so due to frailty, severe comorbidities, or poor performance status, leading to non-cancer competing mortality events that confounded the EFS estimates. Furthermore, the statistical instability arising from such a small denominator means that single events can disproportionately skew survival curves. Therefore, the lack of statistical significance in certain subgroup comparisons should be viewed as a signal of limited power rather than definitive proof of therapeutic equivalence [8,16,44,49].

In conclusion, while this study provides a vital directional framework for precision perioperative therapy—advocating for immunotherapy de-escalation in high responders and intensification in low responders—these results remain hypothesis-generating. The safety of withholding adjuvant therapy in MPR patients and the efficacy of intensification in non-responders must be rigorously validated in large-scale, prospective, randomized clinical trials stratified by pathological response before these strategies can be universally adopted into standard clinical practice.

## 5. Conclusions

In conclusion, this study identifies the pathological response rate (PRR) as a critical predictive biomarker that modulates the therapeutic benefit of postoperative adjuvant therapy in resectable NSCLC. Our findings demonstrate a distinct “gradient of benefit”: patients achieving a Major Pathological Response (MPR) exhibit excellent survival outcomes regardless of postoperative treatment, suggesting that adjuvant immunotherapy might be safely de-escalated or omitted in this favorable subgroup to minimize overtreatment. Conversely, patients with limited pathological response (PRR 0–60%) face a higher risk of recurrence and derive statistically significant survival benefits from intensified adjuvant immunochemotherapy compared to chemotherapy alone or observation. These data challenge the current “one-size-fits-all” approach and support a precision, response-adapted strategy—advocating for intensification in low responders and potential de-escalation in high responders—which warrants further validation in prospective, randomized clinical trials.

## Figures and Tables

**Table 1:** Baseline characteristics stratified by pathological response.

Variable	Total	PRR (0–60%)	PRR (60–90%)	MPR	*p* Value
**Age, y, median (IQR)**	66.0 (60.0–71.0)	65.0 (59.0–69.5)	66.0 (63.0–69.5)	68.0 (60.5–72.5)	**0.428**
**Sex, *n* (%)**					**0.034**
Male	85 (81.0%)	42 (76.4%)	10 (66.7%)	33 (94.3%)	
Female	20 (19.0%)	13 (23.6%)	5 (33.3%)	2 (5.7%)	
**Histology, *n* (%)**					**0.522**
Adenocarcinoma	28 (26.7%)	17 (30.9%)	4 (26.7%)	7 (20.0%)	
Squamous cell carcinoma	77 (73.3%)	38 (69.1%)	11 (73.3%)	28 (80.0%)	
**Tumor size, *n* (%)**					**0.668**
≤4 cm	17 (16.2%)	7 (12.7%)	4 (26.7%)	6 (17.1%)	
4–7 cm	61 (58.1%)	35 (63.6%)	7 (46.7%)	19 (54.3%)	
>7 cm	27 (25.7%)	13 (23.6%)	4 (26.7%)	10 (28.6%)	
**cN, *n* (%)**					**0.177**
N0	32 (30.5%)	17 (30.9%)	6 (40.0%)	9 (25.7%)	
N1	24 (22.9%)	17 (30.9%)	1 (6.7%)	6 (17.1%)	
N2	49 (46.7%)	21 (38.2%)	8 (53.3%)	20 (57.1%)	
**Clinical stage, *n* (%)**					**0.283**
IB	7 (6.7%)	4 (7.3%)	2 (13.3%)	1 (2.9%)	
IIA	10 (9.5%)	4 (7.3%)	0 (0.0%)	6 (17.1%)	
IIB	25 (23.8%)	17 (30.9%)	3 (20.0%)	5 (14.3%)	
IIIA	39 (37.1%)	20 (36.4%)	5 (33.3%)	14 (40.0%)	
IIIB	24 (22.9%)	10 (18.2%)	5 (33.3%)	9 (25.7%)	

**Table 2:** Neoadjuvant regimens and perioperative outcomes for all enrolled patients.

Variable	Total (*n* = 105)	PRR (0–60%) (*n* = 55)	PRR (60–90%) (*n* = 15)	MPR (*n* = 35)	*p* Value
**Neoadjuvant regimen, *n* (%)**					**0.048**
Chemoimmunotherapy	86 (81.9%)	41 (74.5%)	12 (80.0%)	33 (94.3%)	
Chemotherapy	19 (18.1%)	14 (25.5%)	3 (20.0%)	2 (5.7%)	
**Neoadjuvant cycles, *n* (%)**					**0.093**
1	7 (6.7%)	5 (9.1%)	1 (6.7%)	1 (2.9%)	
2	39 (37.1%)	16 (29.1%)	4 (26.7%)	19 (54.3%)	
3	48 (45.7%)	29 (52.7%)	6 (40.0%)	13 (37.1%)	
≥4	11 (10.5%)	5 (9.1%)	4 (26.7%)	2 (5.7%)	
**Surgical procedure, *n* (%)**					**0.223**
Pneumonectomy	7 (6.7%)	6 (10.9%)	1 (6.7%)	0 (0.0%)	
Complex resection	17 (16.2%)	9 (16.4%)	2 (13.3%)	6 (17.1%)	
Wedge resection	1 (1.0%)	1 (1.8%)	0 (0.0%)	0 (0.0%)	
Lobectomy	71 (67.6%)	34 (61.8%)	9 (60.0%)	28 (80.0%)	
Vascular sleeve resection	2 (1.9%)	2 (3.6%)	0 (0.0%)	0 (0.0%)	
Sleeve resection	7 (6.7%)	3 (5.5%)	3 (20.0%)	1 (2.9%)	
**Surgical approach, *n* (%)**					0.323
Open	3 (2.9%)	1 (1.8%)	1 (6.7%)	1 (2.9%)	
VATS	81 (77.1%)	41 (74.5%)	10 (66.7%)	30 (85.7%)	
Conversion	21 (20.0%)	13 (23.6%)	4 (26.7%)	4 (11.4%)	
**ypT, *n* (%)**					0.250
T1	40 (38.1%)	17 (30.9%)	6 (40.0%)	17 (48.6%)	
T2	0 (0.0%)	0 (0.0%)	0 (0.0%)	0 (0.0%)	
T3	13 (12.4%)	7 (12.7%)	3 (20.0%)	3 (8.6%)	
T4	6 (5.7%)	2 (3.6%)	2 (13.3%)	2 (5.7%)	
**ypN, *n* (%)**					**0.646**
NO	62 (59.0%)	29 (52.7%)	9 (60.0%)	24 (68.6%)	
N1	19 (18.1%)	12 (21.8%)	3 (20.0%)	4 (11.4%)	
N2	24 (22.9%)	14 (25.5%)	3 (20.0%)	7 (20.0%)	
N3	0 (0.0%)	0 (0.0%)	0 (0.0%)	0 (0.0%)	
**ypTNM, *n* (%)**					**0.411**
IA	23 (21.9%)	10 (18.2%)	2 (13.3%)	11 (31.4%)	
IB	22 (21.0%)	10 (18.2%)	3 (20.0%)	9 (25.7%)	
IIA	5 (4.8%)	5 (9.1%)	0 (0.0%)	0 (0.0%)	
IIB	22 (21.0%)	11 (20.0%)	5 (33.3%)	6 (17.1%)	
IIIA	33 (31.4%)	19 (34.5%)	5 (33.3%)	9 (25.7%)	
IIIB	0 (0.0%)	0 (0.0%)	0 (0.0%)	0 (0.0%)	
IIIC	0 (0.0%)	0 (0.0%)	0 (0.0%)	0 (0.0%)	
**Pathologic T downstaging,**	***n* (%)**				0.797
Yes	58 (55.2%)	29 (52.7%)	8 (53.3%)	21 (60.0%)	
No	47 (44.8%)	26 (47.3%)	7 (46.7%)	14 (40.0%)	
**Nodal downstaging, *n* (%)**					0.099
Yes	40 (38.1%)	16 (29.1%)	6 (40.0%)	18 (51.4%)	
No	65 (61.9%)	39 (70.9%)	9 (60.0%)	17 (48.6%)	
**Adjuvant therapy, *n* (%)**					0.326
Chemoimmunotherapy	67 (63.8%)	32 (58.2%)	9 (60.0%)	26 (74.3%)	
Chemotherapy	15 (14.3%)	8 (14.5%)	4 (26.7%)	3 (8.6%)	
No adjuvant therapy	23 (21.9%)	15 (27.3%)	2 (13.3%)	6 (17.1%)	

## Data Availability

The original contributions presented in this study are included in the article/Supplementary Material. Further inquiries can be directed to the corresponding author.

## Supplementary Materials

The following supporting information can be downloaded at: https://www.mdpi.com/article/10.3390/cancers18060955/s1, Figure S1. Multivariate Cox Analysis of DFS in a Homogenized Cohort. To minimize clinical heterogeneity, this analysis was restricted to a homogenized cohort of patients with clinical stage II or higher (cStage ≥ II) who underwent definitive anatomical lung resection (lobectomy or pneumonectomy). The forest plot demonstrates that while pathological stage ypIIA was significantly associated with increased recurrence risk (HR = 7.84, *p* = 0.032), Adjuvant IO + Chemotherapy showed a favorable trend toward risk reduction (HR = 0.43, 95% CI: 0.17–1.10). These findings suggest that in a standardized surgical population, intensified adjuvant therapy and major pathological response (MPR) are key independent indicators of improved long-term prognosis. Figure S2. Kaplan-Meier Estimates of DFS Stratified by Treatment and Response. Survival trajectories were analyzed in the homogenized cohort (cStage ≥ II, anatomical resection) to evaluate the synergy between pathological response and adjuvant intensity. A significant difference in DFS was observed across the four subgroups (Log-rank *p* = 0.011), with the “MPR + Adjuvant IO + Chemo” group exhibiting the most superior survival plateau. Conversely, the “Non-MPR + Adjuvant Chemo” group demonstrated the poorest outcomes, confirming that for patients undergoing standardized radical surgery, the combination of achieving MPR and receiving intensified adjuvant immunotherapy is essential for maximizing disease-free survival.
