# Supplementary figures and images for: Response-Adapted Benefit of Postoperative Adjuvant Therapy Following Neoadjuvant Treatment in Resectable NSCLC: A Single-Center Retrospective Cohort Study
Source: Cancers (Basel). 2026 Mar 15;18(6):955. doi: 10.3390/cancers18060955 (PMC13025113; doi:10.3390/cancers18060955)

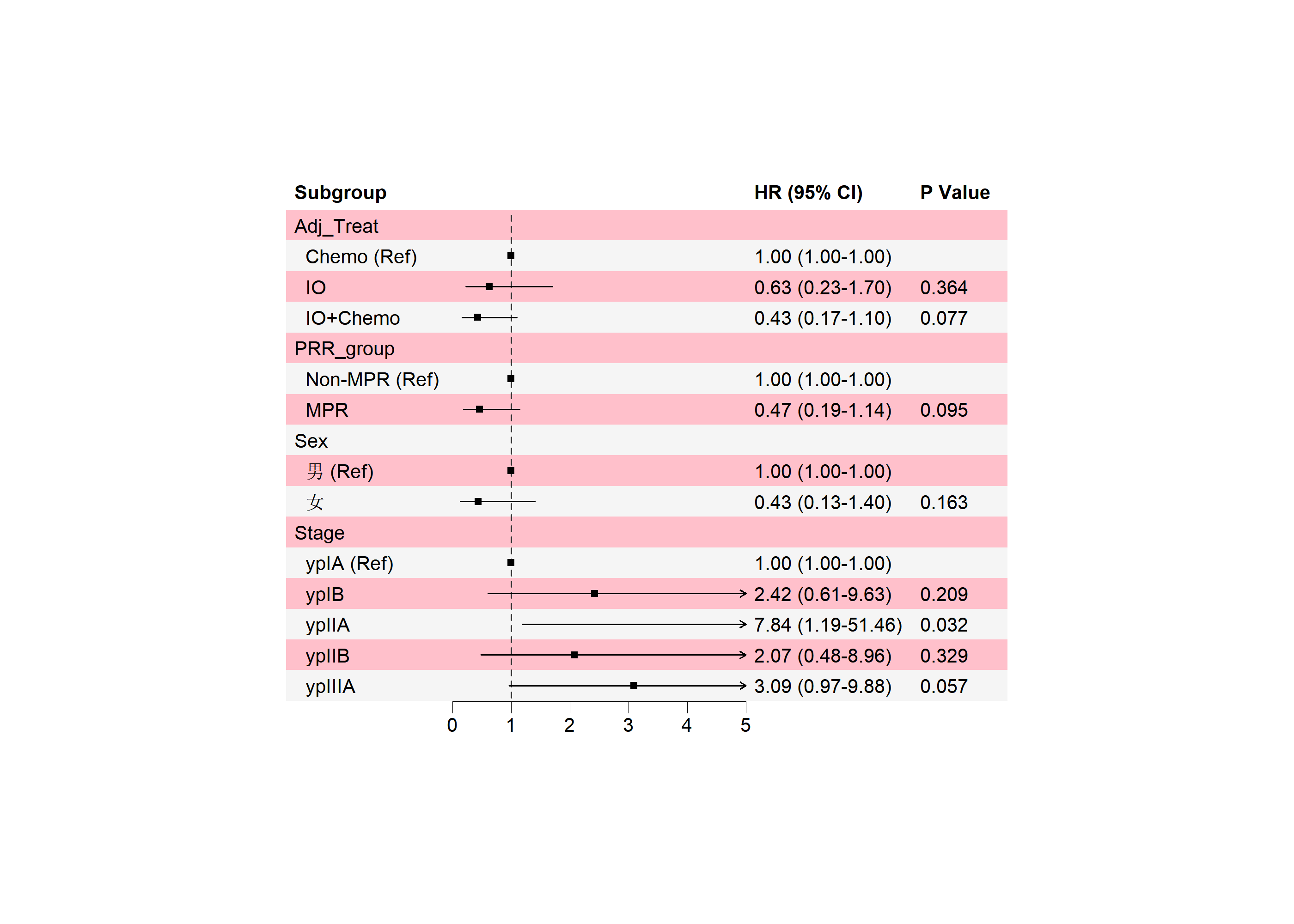

Supplement: Supplementary file 1 [file cancers-18-00955-s001.zip › figureS1.png]

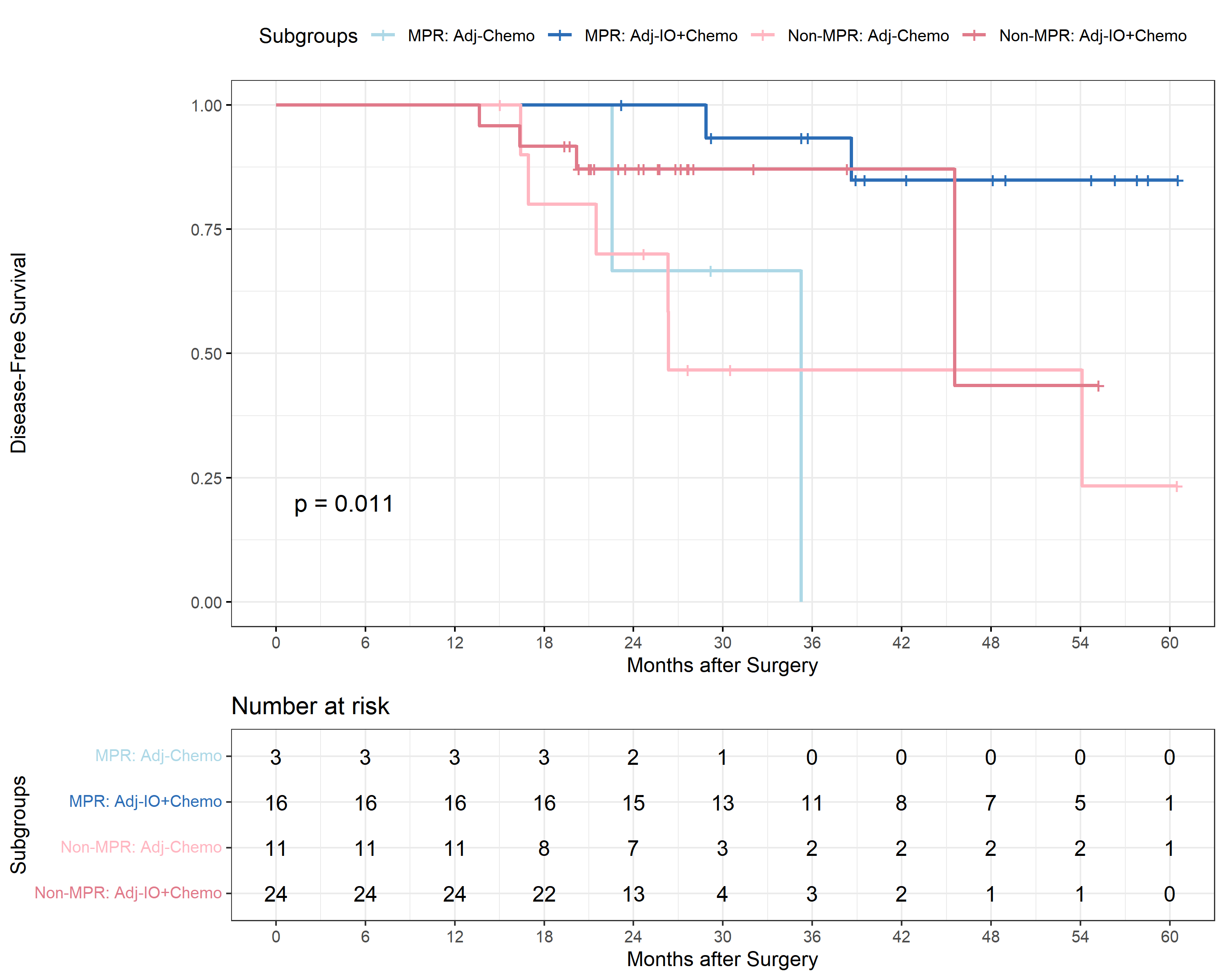

Supplement: Supplementary file 1 [file cancers-18-00955-s001.zip › figureS2.png]
